# Disruptive Behaviors and Intellectual Disability: Creating a New Script

**DOI:** 10.3389/fpsyt.2022.851490

**Published:** 2022-07-08

**Authors:** Melvin Chin-Hao Chan, Mackenzie Campbell, Nadia Beyzaei, Sylvia Stockler, Osman S. Ipsiroglu

**Affiliations:** ^1^H-Behaviours Research Lab, BC Children's Hospital Research Institute, Vancouver, BC, Canada; ^2^Division of Biochemical Diseases, Department of Pediatrics, Faculty of Medicine, The University of British Columbia, Vancouver, BC, Canada; ^3^Divisions of Developmental Pediatrics, Respirology, and Child and Adolescent Psychiatry, Departments of Pediatrics and Psychiatry, Faculty of Medicine, The University of British Columbia, Vancouver, BC, Canada

**Keywords:** intellectual disability, Down syndrome, disruptive behavior, sleep, physical activity

## Abstract

**Background:**

Terms currently used to describe the so-called challenging and disruptive behaviors (CBDs) of children with intellectual disabilities (ID) have different connotations depending on guiding contextual frameworks, such as academic and cultural settings in which they are used. A non-judgmental approach, which does not attempt to establish existing categorical diagnoses, but which describes in a neutral way, is missing in the literature. Therefore, we tried to describe CDBs in youth with ID in an explorative study.

**Methods:**

Interviews with families investigated the CDBs of five youth with Down syndrome. At home, families tracked youth's sleep/wake behaviors and physical activity. Youth were observed in a summer school classroom. The collected information and suggested explanatory models for observed CDBs were reviewed with the families.

**Results:**

We grouped CDBs as *challenging*, if they were considered to be reactive or triggered, or *unspecified*, if no such explanatory model was available. A third category was created for light-hearted CDBs: *goofy*, acknowledging the right to laugh together with peers. We found some relationships between sleep, physical activity, and CDBs and developed an explorative approach, supporting a child-centered perspective on CDBs.

**Conclusion:**

The controversial discussions on terminology and management of CDBs in the literature demonstrate the need for a non-judgmental approach. Such an explorative approach, allowing non-professionals to not label, has been missing. The fact that, up to now, the light-hearted behaviors of an individual with ID have not been integrated in commonly-used behavioral checklists as their natural *right*, proves our concept and indicates that a paradigm change from judgment-based to exploratory-driven approaches is needed.

## Introduction

Although it is common practice to assess contributing and trigger factors of challenging and/or disruptive behaviors (CDBs) in children with intellectual disability (ID) (1), the terms used to describe these behaviors (2) have different connotations depending on guiding contextual frameworks, such as the academic and cultural settings (3, 4) in which they are used. For example, problem behavior (5, 6) and challenging behavior (4, 7) are used interchangeably and focus on deviations from conventional social norms and inability to access services. Social, emotional, and behavioral difficulties (8) and disengaged, delinquent, and troubled and troubling behaviors (9) carry different, mainly negative, connotations. Terms for behaviors that may cause concern are shown in Table 1. The lack of harmonization may be due to the absence of a shared language among medical and educational professionals, parents who provide lifelong care, as well as individuals with ID themselves (11, 12). In the medical literature (13–15), CDBs is the terminology used. Coming from a clinical background and trying to understand decision-making in a school setting—which is usually influential for parental and clinical decision-making—we use CDBs as it is consistent with the medical and educational systems in our geographical context of British Columbia, Canada. However, which behaviors are connoted as challenging and/or disruptive and who decides that? What are the contributing factors to these CDBs? It is important to be aware of and reflect on these questions because answers result in different models used to manage CDBs (16–20).

**Table 1:** Terms for concerning behaviors.

**Term**	**Elaboration**	**Discipline(s)**
Problem behavior	“The problem behavior results in a significant negative impact on the person's quality of life or the quality of life of others. This may be owing to restriction of his or her lifestyle, social opportunities, independence, community integration, service access or choices, or adaptive functioning” OR “The problem behavior presents significant risks to the health and/or safety of the person and/or others.” [(10) p84].	Psychology, psychiatry
Challenging behavior	“Culturally abnormal behavior(s) of such an intensity, frequency or duration that the physical safety of the person or others is likely to be placed in serious jeopardy, or behavior which is likely to seriously limit use of, or result in the person being denied access to, ordinary community facilities” (4 p3).	Psychology, psychiatry, medicine, health
Social, emotional, and behavioral difficulties	“While there is no standard definition of [social, emotional, and behavioral difficulties], the various definitions share commonalities such as the following: behavior that goes to an extreme; behaviors or emotions that are outside societal norms; behaviors or emotions that negatively affect a child's educational functioning” (8 p276).	Psychology, education
Disengaged, delinquent, troubled, and troubling behavior	“Labels such as ‘disaffected', ‘disengaged', ‘disruptive', ‘delinquent', ‘challenging', ‘troubled and troubling' and disorders including ADHD, Oppositional Defiant Disorder and Conduct Disorder, all have a degree of overlap with [social, emotional, and behavioral difficulties] in terms of external behavior” (9 p97).	Psychology, education
Disruptive behavior	“Oppositionality, conduct problems, or aggression” (14 p65).	Medicine, psychology, education

Therefore, we investigated the CDBs of youth with ID and explored natures and possible triggers of these behaviors in individuals with Down syndrome. We used a grounded theory methodology, as applied in developmental pediatrics and child psychiatry (21) and tried to review each behavior's meaning from the perspective of the participants using *in dubio pro reo* (in cases of doubt, then for) (22). Down syndrome is a chronic, complex condition with multiple comorbidities (23–25), with ID as a common denominator. Depending on the background of the authors, individuals with Down syndrome are reported to have a range of CDBs (26, 27), but few researchers have investigated their natures and etiologies (26, 28). Mimicking the coining decision-making in the community, we focused on reported and observed CDBs at home (29–31) and at school (32). In addition, we explored lifestyle factors—specifically, physical activity and sleep—and parental perceptions to understand CDBs in everyday lives.

## Methods

We partnered with the Down Syndrome Resource Foundation (DSRF; Burnaby, Canada; www.dsrf.org) for this exploratory study. The DSRF provides resources and services (e.g., library, math instruction, speech-language therapy) “to empower individuals with Down syndrome to reach their full potential” (33). The study took place at the DSRF's summer school program for individuals with Down syndrome aged 10- to 20-years-old in Summer 2016 (http://www.dsrf.org/media/Summer%20School%20FINAL%202016.pdf). Individuals with Down syndrome could attend one or more sessions of the summer school, each lasting two weeks. The daily schedule was: (a) morning reading class (1.5 h), (b) snacktime (15 min), (c) morning math class (1.5 h), (d) lunch (45 min), and (e) afternoon class (2 h)—either art and hip hop dance or Bollywood dance and yoga, depending on session's theme. The study's concept and methodology was developed in consultation with parents and staff at the DSRF and peer-reviewed by parents and professionals not involved in the study. Research ethics approval was obtained from The University of British Columbia (H16-01280).

### Participants

Individuals with Down syndrome who were attending the summer school and whose parents/caregivers reported *day- and/or night-time challenging and/or disruptive behaviors* were eligible for participation per an email advertisement. The advertisement did not further specify the terms *challenging and/or disruptive* and we left the interpretation to parents/caregivers. As our intent was observation, no formal medical assessments were done. However, participants' medical backgrounds were discussed during the interviews and summary recommendations were made at the end of the study.

Of the 50 families whose children attended the summer school, five consented to participate. The median age of participants was 13 years (*M* = 12.8, *SD* = 0.57, range = 12–14). Four individuals were male. All individuals had sleep problems and were waiting for a sleep assessment or the next step of sleep medicine-related therapeutic interventions. Table 2 presents vignettes for all participants. P5 had very severe CDBs (including on the first day of the summer school) and was not permitted to continue attending. He and his family remained enrolled in the study and participated in data collection, except for observations. All other participants attended the summer school and had an assigned educational assistant to support them throughout the program.

**Table 2:** Participant vignettes.

	**P1**	**P2**	**P3**	**P4**	**P5**
**Demographics**	13 years old; male.	13 years old; male.	12.5 years old; female.	13.5 years old; male.	12 years old; male.
**Diagnosis**	Selective mutism, status post infantile spasms, sensory processing dysfunctions.	Autism spectrum disorder, iron deficiency, tics, status post tonsillectomy/ adenoidectomy, sensory processing dysfunctions.	Status post tonsillectomy/ adenoidectomy, familial restless legs syndrome (RLS), sensory processing dysfunctions.	Status post tonsillectomy/ adenoidectomy, overweight, sensory processing dysfunctions.	Possible ADHD, autism spectrum disorder (under investigation), sensory processing dysfunctions.
**CDBs**	Sudden withdrawals, anxiety, ‘goose-step' marching.	Less focused on academics & favors physical activities, always wants to be in a group, anxiety (sudden withdrawals and shutdowns), fidgety, stubborn, “flips” from activity to activity.	Stubborn, “not look at you and walk away” if she does not want to interact or participate, frequent temper tantrums.	‘Class clown' behaviors (described as familial), difficulty regulating emotions, “freaks out” (emotional pain, “collapsing in”), impulse control, defiant, makes “weird” sounds, picks nose.	Fidgety, self-stimulation with paper, copies others, occasionally aggressive, rude and/or disengaged.
**Observations**	Average inter-observer reliability (Cohen's kappa): 0.927.	Average inter-observer reliability (Cohen's kappa): 0.874.	Average inter-observer reliability (Cohen's kappa): 0.989. Did not have any goofy behaviors in the school setting, which became a concern for us at the end of the observation period because P3's parent reported goofiness at home.	Average inter-observer reliability (Cohen's kappa): 0.997.	Had very difficult behaviors on the first day & was not allowed to attend; general observations about the character and severity of CDBs were recorded by his mother at home.
**Physical activities**	Special Olympics (bowling), dance, Taekwondo.	Special Olympics, swimming, tennis, walking.	Walking, hiking, “chasing game”.	Walking, basketball, swimming.	Walking, running, basketball, swimming, biking.
**Sleep problems**	Insomnia (nighttime awakenings, early morning awakenings), difficulty breathing, non-restorative sleep (restless sleeper); family history of insomnia (mother & sister).	Insomnia (nighttime awakenings, early morning awakenings), difficulty breathing, non-restorative sleep (restless sleeper); family history of insomnia (mother).	Insomnia (nighttime awakenings, early morning awakenings), non-restorative sleep (restless sleeper); family history of insomnia (mother).	Insomnia (previously; nighttime awakenings, early morning awakenings), nightmares/“horrors”, non-restorative sleep (restless sleeper); family history of insomnia (mother).	Insomnia (falling asleep problems, nighttime awakenings), occasional major hyperactivity before bedtime with bedtime resistance, non-restorative sleep (restless sleeper); family history of insomnia (mother & sisters, if no physical activity during daytime).
**Physical activity/sleep interactions**	More CDBs after a poor quality sleep and fewer CDBs after a good-quality sleep. Physical activity may increase sleep quality, which may reduce CDBs the next day.	May have fewer CDBs after receiving physical activity and a good-quality sleep. Family also sees a difference in behavior after 3–4 days of inconsistent/little sleep.	Physical activity may help her go to bed; however, it is difficult to determine the effect of daily physical activity and sleep quality on CDBs, as she did not receive much daily physical activity (lowest among all participants) and her sleep quality was consistently “poor”.	May have more CDBs after shorter sleeps. Physical activity may have a positive effect on his sleep quality. However, it is difficult to determine the effect of daily physical activity and sleep quality on CDBs, as he receives near daily physical activity and his sleep quality was consistently “very good”.	Physical activity may have a positive effect on his sleep quality, but did not seem to affect the occurrence of CDBs. However, P5 may have more CDBs following a lower-quality sleep.

*Families reported demographic information, diagnoses, challenging and/or disruptive behaviors, physical activities, and sleep problems. Research assistants observed Participants 1–4 during summer school; Participant 5 was observed by his family at home. The interaction between physical activity and sleep was interpreted together by the research team and reported to families. Original quotations from the parents are indicated with quotation marks. CDBs, Challenging and/or disruptive behaviors*.

### Data Collection

#### Observations

Two research assistants (RAs) independently observed and recorded the CDBs of four participants during the summer school using the partial interval recording method (34). To become familiar with the partial interval recording method, RAs completed two 2-h training sessions with an experienced DSRF staff member. The observers were not explicitly asked to reflect or document the emotions that CDBs generated in themselves, but this was discussed during the team reviews of the descriptions and codes. During the study period, RAs observed each participant for 20 s per min for 20 min at the beginning, middle, and end of each class (total of 180 min of observations over nine periods per day). A brief description accompanied each observed CDB. Participants were not observed for the remaining 40 s of each min. To become familiar with individual behaviors of each participant, the first day of observations was considered a trial and the research team reviewed the observed behaviors and the contexts in which they occurred to develop a shared language and approach for future observations. Participants were observed for four to five days in the classroom, depending on their attendance at the summer school. Inter-observer reliability (Cohen's kappa) across all observations and all participants was 0.945, indicating high agreement on identifying CDBs in the classroom.

#### Interviews

Individual intake interviews and exit interviews aimed, respectively, to explore individual day- and night-time behaviors and to summarize and share individual findings and counsel families regarding probable next steps. Core elements of all interviews were explorative semi-structured interviewing for the creation of emplotted narratives utilizing empathy and non-judgmental language to understand familial explanatory models. Explorative semi-structured interviewing employed ethnography with open-ended questions (3, 35) to characterize identified CDBs. Emplotted narratives (36) were created by encouraging parents to describe, in their own words, the sleep/wake-related behaviors of their child in the context of everyday routines and by collaboratively co-constructing the investigated history in a plot-like scenario using visualizing descriptions (21). Empathy involved putting ourselves in the place of another in order to reduce bias (37). In all interviews, special emphasis was given to the exploration of transitioning situations at day-, bed-, and night-times. Video clips of CBDs to provide a deeper understanding of CBDs were described by families verbally, but could not be used in our study due to privacy concerns.

#### Daily Diary and Log

Over a two-week period, each participant's family completed a daily diary and log that asked about the participant's: (a) amount of physical activity and nighttime sleep received the day/night before; (b) perceived daytime and nighttime challenges the day/night before; and (c) sleep quality (on a 5-point Likert scale), as assessed first thing in the morning based on the participant's mood and how refreshed they presented. The daily diary and log took ~3–5 min to complete and could be completed on paper forms or via the web [see Heng et al., (38), for information about the web version]. Daily text message reminders were offered to families to complete the diaries and logs; one family requested reminders, which were sent and received during the study period.

### Data Analysis

We utilized ethnographic exploration and empathy (3, 21, 37) as the foundational approach to data analysis to understand the triggers of observed CDBs. First, similar descriptions of behaviors were grouped to generate initial codes, and new descriptions were continually tested against the initial codes to revise the coding scheme. Second, we periodically met as a research team to analyze and review the descriptions and codes together. During the review process, the interviews and daily diaries and logs were used to contextualize the descriptions and generated codes. Descriptive statistics were used to explore the effect of sleep and physical activity on CDBs (e.g., determine whether participants had fewer CDBs after a higher quality sleep). Due to the small sample size, inferential statistical analyses were not conducted.

## Results

**Figure 1:**
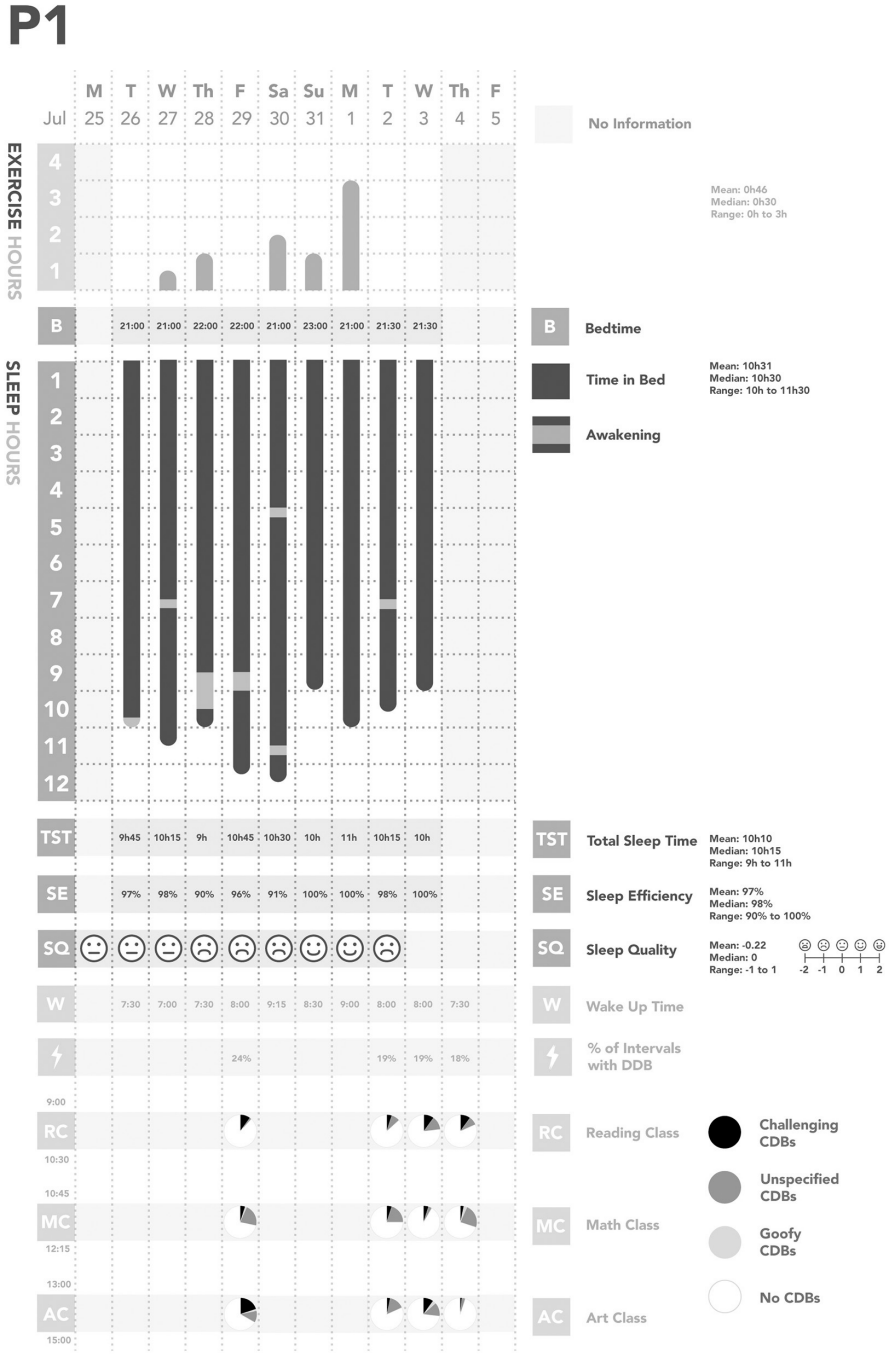
Summary of Participant 1 (P1)'s data. CDBs, Challenging and/or disruptive behaviors.

**Figure 2:**
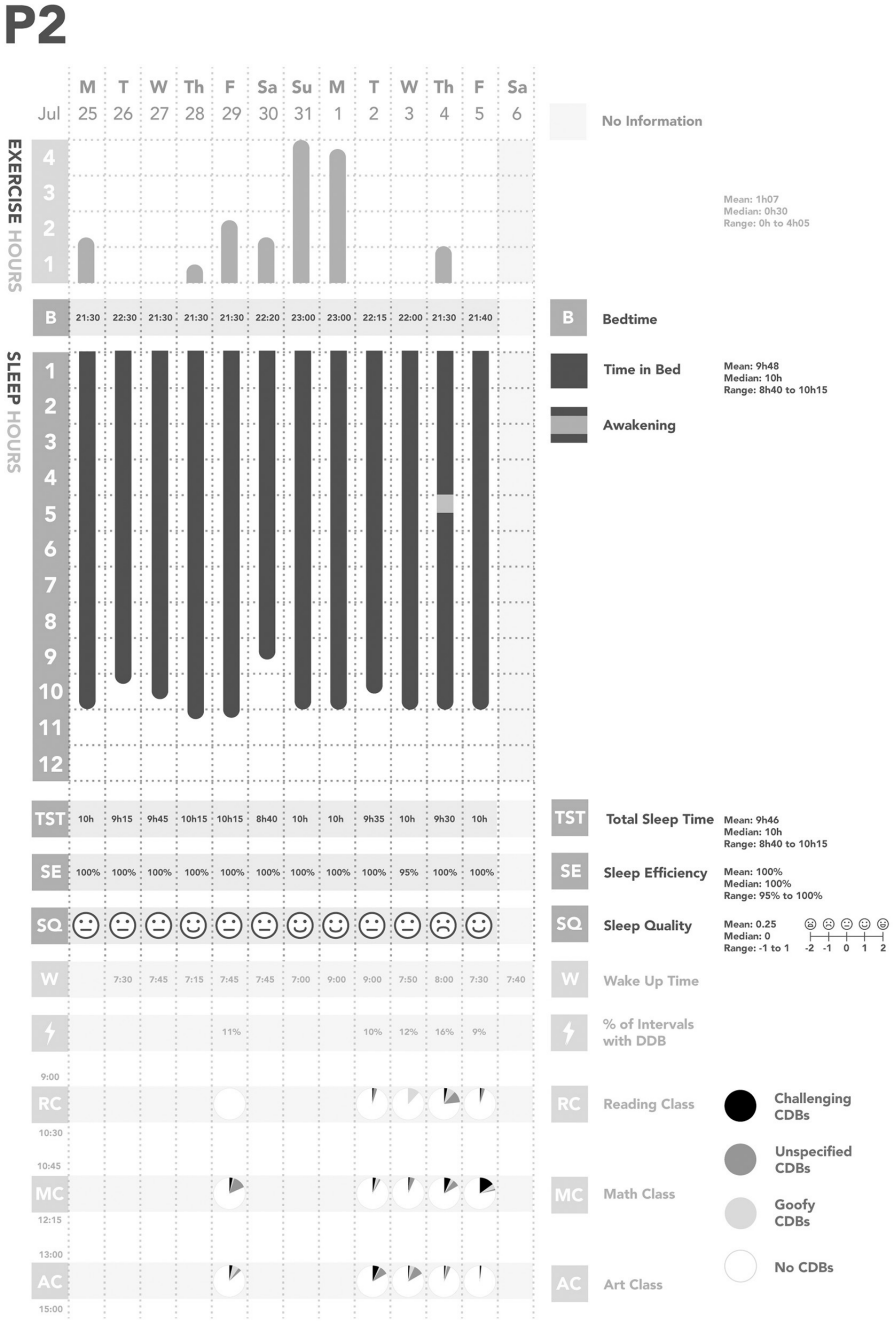
Summary of Participant 2 (P2)'s data. CDBs, Challenging and/or disruptive behaviors.

**Figure 3:**
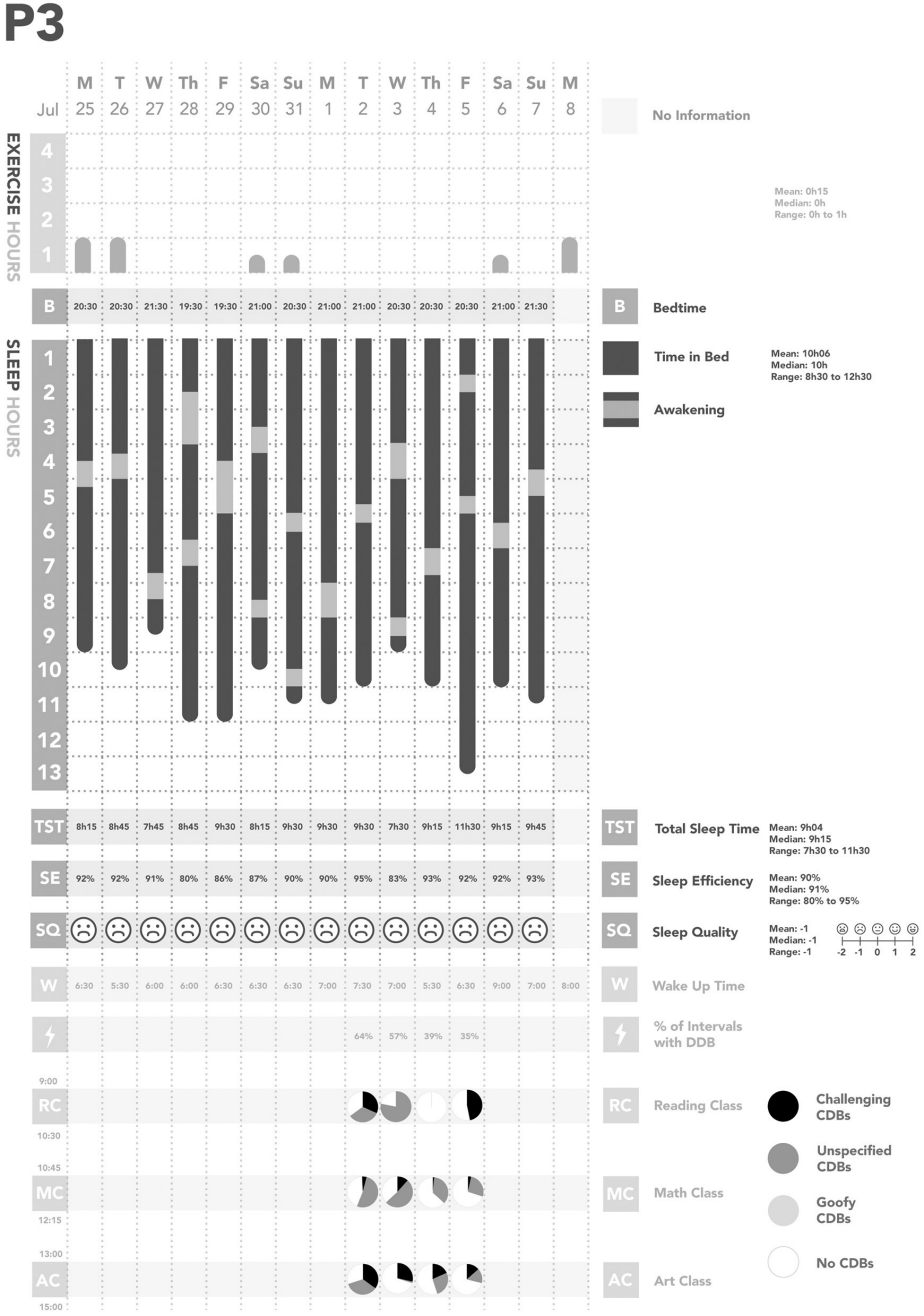
Summary of Participant 3 (P3)'s data. CDBs, Challenging and/or disruptive behaviors.

**Figure 4:**
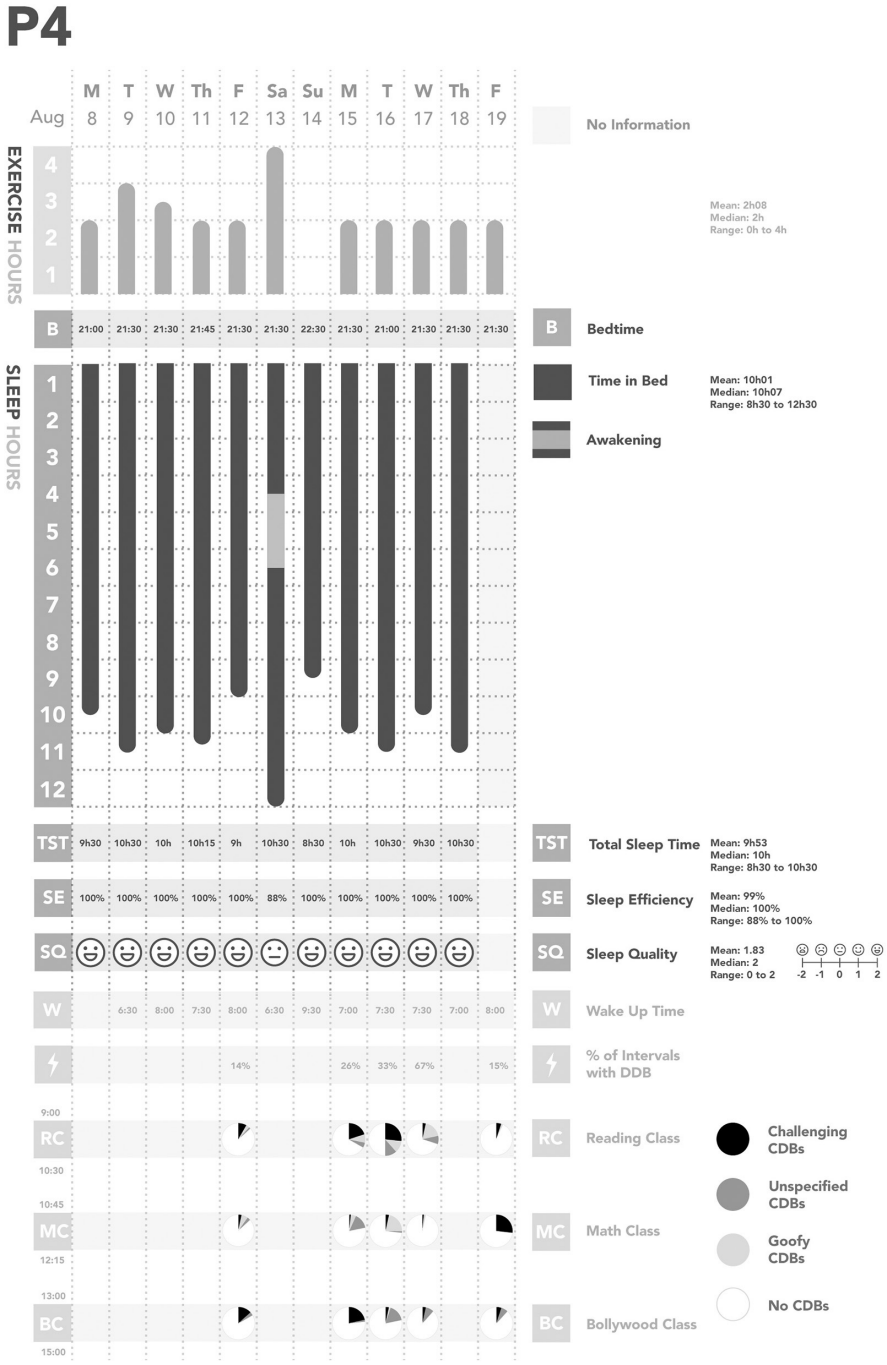
Summary of Participant 4 (P4)'s data. CDBs, Challenging and/or disruptive behaviors.

**Figure 5:**
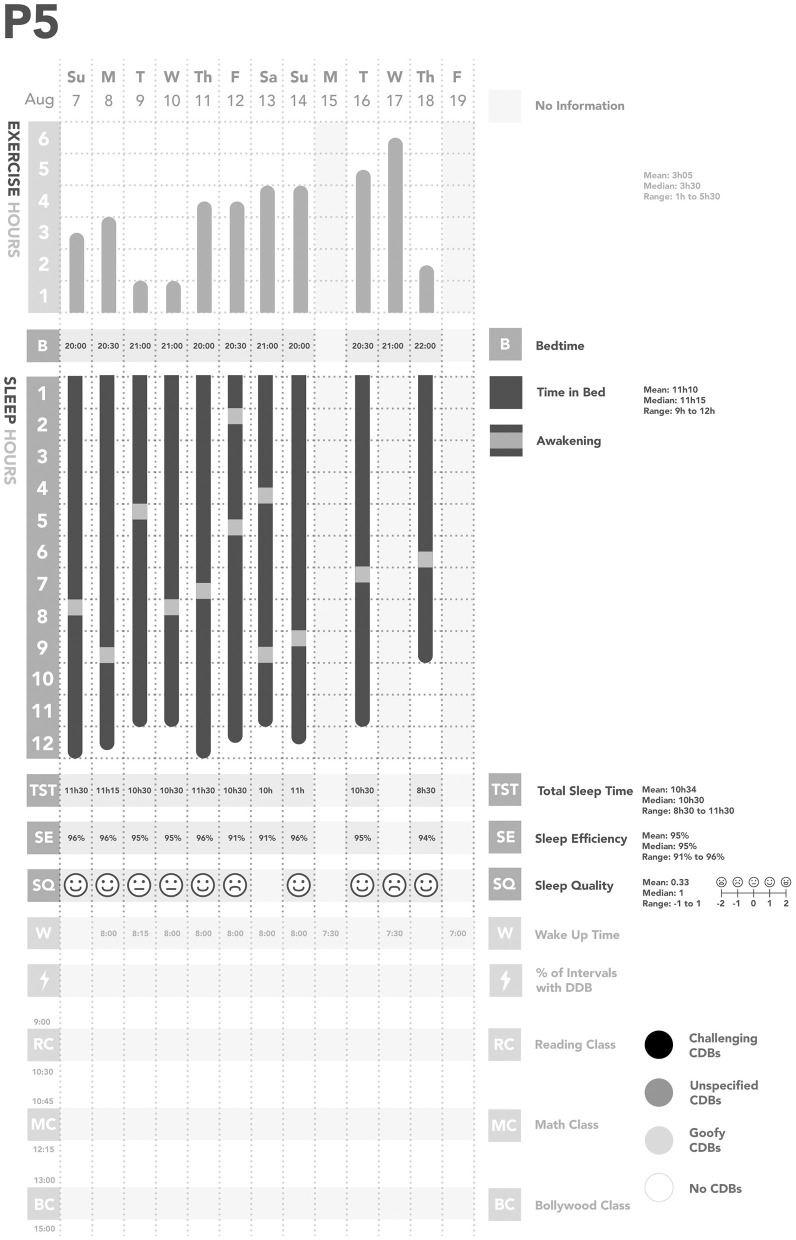
Summary of Participant 5 (P5)'s data. CDBs, Challenging and/or disruptive behaviors.

Across all days of observations, each participant was observed to have their own individual pattern of CDBs (Figures 1–5). In considering the possible origin of each CDB, three categories emerged (Table 3): *challenging, unspecified*, and *goofy*. In the following sections, each CDB is listed and explained with descriptions and/or quotations from parents. From the available information about lifestyle factors, we found that physical activity and sleep may have affected the occurrence of CDBs.

**Table 3:** Categories of challenging and/or disruptive behaviors.

**Challenging**	**Unspecified**	**Goofy**
*Characteristics of temper tantrums:* Outbursts, such as crying, screaming, throwing objects, and pouting	*Disengaging:* Suddenly separating oneself from an activity for no apparent reason; shutting down	*Class clown:* Behaviors were those that were intended to make others laugh
*Stubbornness:* Unwillingness to move on from the current activity to a new task	*Not doing as told:* Refusing to respond to a request	*Distracted:* Distracting themselves with activities or behaviors that may be more fun or sensory seeking
*Being overly enthusiastic:* Interjecting when it is not their turn; speaking louder than others to be the central focus	*Intrusive:* Invasion of someone else's personal space	
*Being self-conscious:* Overly aware of oneself; discomfort in a situation where focus may be directed toward the individual	*Self-stimulation or stimming:* Behaviors that were intended to soothe oneself	

### Challenging Behaviors

*Challenging* behaviors responded to triggers. There were four types of *challenging* behaviors: characteristics of temper tantrums, stubbornness, being overly enthusiastic, and being self-conscious. All participants had *characteristics of temper tantrums*, which were often triggered by unpleasant experiences or situations. Examples include:

- ***Mild:*** *dismissing an educational assistant during an explanation and saying to an educational assistant, “You*'*re getting nothing! Don*'*t touch it!”*
- ***Moderate****: hitting an educational assistant and kicking a backpack*.
- ***Extreme:*** *cascade — First, P4 ran out of the room and into the stairwell; his educational assistant followed. Then, the educational assistant blocked the participant*'*s path and asked him where he was going. He was breathing deeply and tried to calm himself down. After the educational assistant asked for him to return to class, he kicked the educational assistant in the stomach. He seemed shocked at his kicking behavior. The educational assistant told him firmly that it was not okay to kick and tried to bring them back to the classroom. However, he hid in the washroom. Finally, a teacher and the executive director both had to intervene to return him to the classroom*.

*Stubbornness* was observed among all participants. Examples include:

- *Refusing to come back to class (after taking a planned/scheduled break) which was characteristic of P3. The trigger may have been her mother*'*s presence in the building; indeed, her mother noted during the intake interview that P3 will “remove herself from the classroom” and run to her when she does not want to participate in the activity*.
- *Turning one*'*s body away to continue using an iPad when asked to put it away, which may have been triggered by a deep engagement with an activity on the iPad*.
- *Not finishing the snack or lunch on time and then refusing to stop eating because the break is over, which may have been triggered by continued hunger*.

*Being overly enthusiastic* was characteristic of one participant who seemed to be highly interested in most activities during the summer school. The trigger for these behaviors may have been wanting to participate in the activities, but disrupting the teaching flow. Examples include:

- *Interrupting the teacher*,
- *Grabbing a pen from an educational assistant who was explaining the activity, and*
- *Shaking a cue card in someone*'*s face (where the activity involved using the cue card; in this instance, the participant also had a large smile and seemed highly interested in the activity)*.

*Being self-conscious* was observed in only two participants. The trigger for these behaviors may have been difficulties focusing on the task or feeling vulnerable. Examples include:

- *Playing with one*'*s shirt and pants during class*,
- *Wrapping one*'*s arms around oneself, and*
- *Looking around during class*.

### Unspecified Behaviors

*Unspecified* behaviors did not have triggers. There were four types of *unspecified* behaviors: disengaging, not doing as told, intrusive, and self-stimulation or stimming. All participants had *disengaging* and *not doing as told* behaviors, which comprised the majority of *unspecified* behaviors.

- *Examples of “disengaging” are leaving the room suddenly for a break (when the participant was supposed to be in class) and playing with a hat instead of doing work during class*.
- *Examples of “not doing as told” are being uncooperative, not answering a question asked by an educational assistant, refusing to give a book back to an educational assistant by sitting on it, and sitting on the ground instead of participating in the yoga class. One participant also said, “You go, I stay!” to their educational assistant and crossed their arms when asked to stand up and go pretend grocery shopping with the rest of the class*.

All participants also had *intrusive* behaviors, but these occurred infrequently based on classroom and parental observations. Examples include:

- *Pushing an educational assistant*'*s head down toward the table*,
- *Leaning on the teacher instead of working*,
- *Touching or reaching for an educational assistant*'*s face without consent to do so, and*
- *Grabbing items from others*.

Lastly, the only instances of *self-stimulation/stimming* were from P3. P3 often shook her doll toy, sometimes to the point of total distraction.

### Goofy Behaviors

*Goofy* behaviors were developed given our interpretation that participants were having fun and socializing by displaying those behaviors in a protected place among peers, but were also disturbing the flow of the class. These behaviors were categorized as *class clown* or *distracted*.

- *Examples of “class clown” behaviors include pretending to lick an educational assistant, looking at the number line through fingers (like cheating), flicking water onto the table and another student using a paintbrush, and painting on the table instead of the paper*.
- *Examples of “distracted” behaviors are swiveling around in an office chair; hitting a paper worksheet against one*'*s face; and saying “I need your finger please” to a teacher, which was unrelated to the task at hand*.

Although four participants (all boys) had *goofy* behaviors during the summer school, P3 (girl) did not have any.

### Contributing Factors

We also found some relationships between physical activity, sleep, and CDBs (Table 2; Figures 1–5). For all participants, physical activity in the daytime seemed to affect sleep quality on the same night. Higher levels of physical activity seemed to increase sleep quality.

- *For P1 and P2, this increased sleep quality may have resulted in fewer CDBs the following day*.
- *For P5, the trend appeared reversed: less physical activity seemed to lower sleep quality, which increased the occurrence of CDBs the next day*.

Families also perceived links between physical activity, sleep, and CDBs. For example:

- *P2*'*s family reported, “When he is sleep deprived, he is not as compliant, more rigid in thinking and more emotional” and “He flips from activity to activity quicker if he hasn*'*t had enough sleep, [which] may be because he knows that he will ‘drop' if he stops.”*
- *P3*'*s mother reported that “exercise puts her in a state of relaxation and helps her to fall asleep.”*
- *P5*'*s family reported that, “He*'*ll be tired when he*'*s done a lot all day” and that he is a “busy kid [who] usually [goes] to bed really well.”*

## Discussion

**Figure 6:**
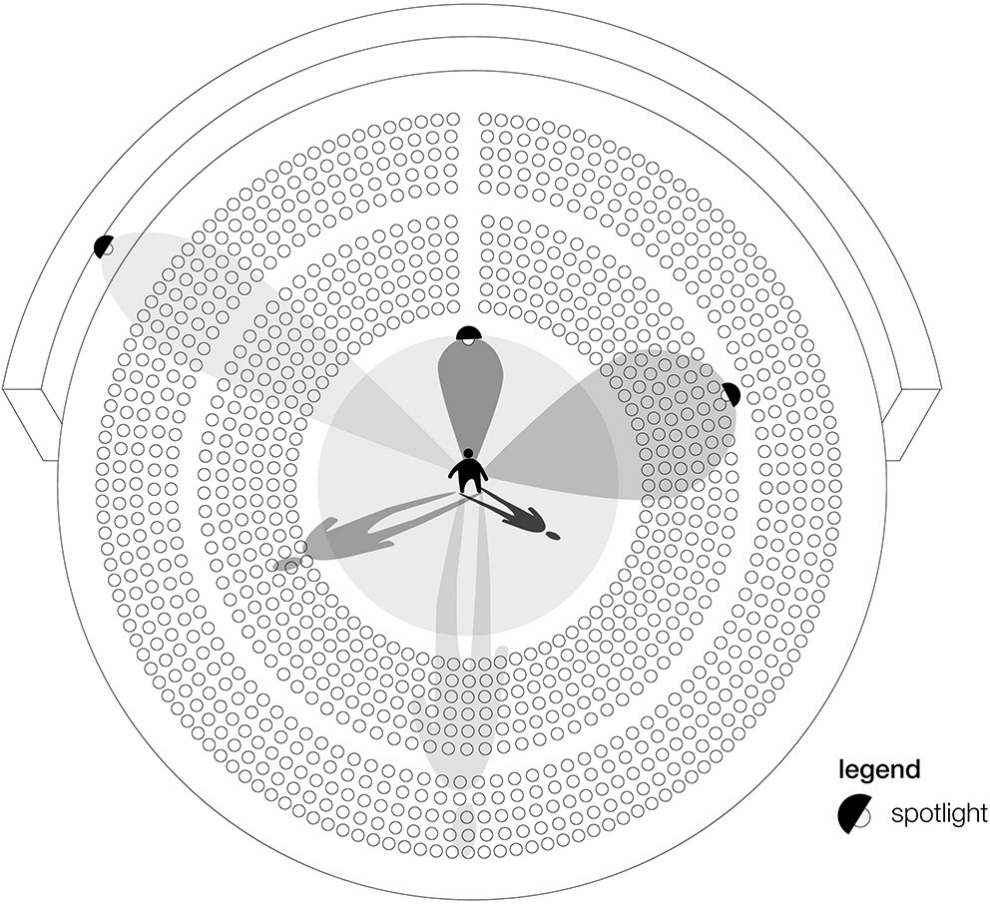
Visualization of our explorative approach. The observed individual is located on the stage and is surrounded by their community (individuals sitting in the theater, including parents, family, healthcare professionals, educators, etc.). Depending on the strength of the spotlight (in our context, training-based rigor), a higher or lower visual acuity might be achieved and depending on the positioning of the spotlights, the dimension of the shadow changes.

Kleinman (35) suggests the utilization of narratives in clinical history-taking to contextualize illness. We adapted this concept for application in the community setting, namely in a summer school setting for individuals with Down syndrome. As a team, we first listened to the parental narratives. Then, we explored and merged our understandings from the narratives with our structured observations. All CDBs were reviewed with visualizing descriptions (like in movie sequences) from the participants' (assumed) perspective using *in dubio pro reo* (in cases of doubt, then for) (22), which in our context meant to not label but describe. We were able to create new narratives as plots or scripts to explain why certain CDBs might have happened, which were shared with the parents and then the summer school team. Mattingly (36) calls the creation of such shared new plots, “therapeutic emplotment” (p811). Our new plots were reviewed and negotiated before presentation among the two independent RAs, with clinicians, and eventually with the parents, and in one case the siblings, of the participants. This exercise resulted in the creation of an explorative approach (Figure 6), which can widen our standardized medical and psychological approaches to CDBs. Traditional approaches use Venn diagrams to visually summarize mutually exclusive and co-existing factors, independent of a formal statistical analysis (39). Our understanding is that the perspective of the viewer affects what the viewer can see (3, 21), and the positioning of the spotlight may change the dimension of the shadow and what is visible and not visible. Moreover, depending on the strength of the spotlight (in our context, training-based rigor), a higher or lower visual acuity might be achieved. Thus, our explorative approach allows us to focus on the observed individual and review their behaviors without connoting them as appropriate or inappropriate, despite the fact that the behaviors may be challenging and/or disruptive for parents and teachers alike.

### Categories of Challenging and/or Disruptive Behaviors

CDBs are constructed (2, 40) and contextual where “the individual shapes his or her environment and in turn is shaped by it” [(41) p228]. In our analysis of observed CDBs, three non-stigmatizing and non-clinical categories emerged to describe the behaviors within the context of a summer school attended by individuals with Down syndrome.

First, *challenging* behaviors, such as being slow, inattentive, or moody, may have a variety of triggers. Although individuals with Down syndrome have deficits in processing speed (42), as autonomous individuals, they may have simply wanted more time to finish eating or to engage in a different activity. There may also have been an extrinsic trigger. For example, in P4's temper tantrum cascade described in the previous section, it surfaced that the educational assistant told the participant that he was untrustworthy and he could not go to the washroom by himself, which may have initiated his *challenging* behavior. *Challenging* behaviors may provoke dialogue to understand what happened and to negotiate future activities. However, if individuals are injuring themselves or endangering others, applied behavior analysis (16, 43) or positive behavioral support (44) could be used to extinguish the *challenging* behaviors and/or implement replacement behaviors.

Second, we interpreted behaviors for which we could not identify specific triggers as *unspecified*. These neutral descriptions signal the need for further assessment and exploration to understand the origins of *unspecified* behaviors. *Unspecified* behaviors may not be inherently problematic. Instead, they invite a review of the setting to determine the extent to which it met the needs of the participants. A biomedical explanation could be slower processing speed or sensory processing abnormalities (45, 46), which may limit one's participation in activities and thus cause emotional vulnerability. For example, during the intake interview, one mother told us that her son during a trip would suddenly sit down in the street, become tearful and say, “It's the Down syndrome way.” Indeed, all five families reported in the intake interviews that feeling overwhelmed was a trigger for their children's CDBs (for P1, this was described in terms of feeling anxious).

Third, *goofy* behaviors interrupted the teaching flow and were thus considered CDBs. Reviewing the contextual framework, however, we realized that *goofy* behaviors were initiated in a social environment with peers and familiar others. According to our observations, they were not purposefully *challenging* or *disruptive*, but light-hearted. During the review of a previous version of this manuscript, a reviewer challenged our category of *goofy* behaviors by asking, “Goofy behaviors, if disruptive, should still be coded as challenging too? Likely have a root cause of avoidance?” Although teachers or parents may view *goofy* behaviors as avoidance or non-compliance, after reviewing the triggers of *goofy* behaviors in the context of being together with peers in long teaching sessions of 90 min, we interpreted them as stemming from a place of being harmlessly silly. Avoidance was not considered as a possible trigger because the interpretation pathologizes CDBs without considering the context and the variety of factors causing laughter, a basic human emotion that promotes learning and creativity (47). Indeed, *goofy* behaviors may not be issues for individuals with Down syndrome themselves, but actually issues for us (including teachers) who wish to maintain a particular flow to satisfy learning standards. Interestingly, P3 was the only participant who did not have any *goofy* behaviors in the classroom setting, despite her mother's description during the exit interview that her daughter loves to joke around when she is at home. Was P3 possibly constantly stressed or just adherent to the rules of the summer school? Upon further inquiry, P3's mother also mentioned that her daughter has a perfectionistic side, which may have influenced her behavior at summer school, going along with a wealth of *challenging* and *unspecified* CDBs.

### Contributing Factors

In addition to the CDB-related considerations, we found some relationships between physical activity, sleep, and CDBs. In one case (P3), physical activity helped the participant to sleep better than usual and be able to fall asleep at the scheduled time. Most participants had higher sleep quality after being physically active during the daytime and, for two participants, this higher sleep quality was associated with fewer CDBs the following day. Further, three families (P2, P4, P5) reported very high levels of daily physical activity. Although a large amount of physical activity could be considered normal, it could also conceal symptoms of medical conditions, such as familial Restless Legs Syndrome (RLS), as is suggested for other patient groups (48, 49). RLS is a sensorimotor neurologic disorder causing sensory seeking behaviors (50). The presence of insomnia and positive family history among participants suggested that these individuals may be on the RLS-spectrum; we assumed that the sensorimotor discomfort was reduced by physical activity. Thus, physical activity may be an important factor to investigate in relation to CDBs.

In addition to RLS, sleep-disordered breathing is highly prevalent in individuals with Down syndrome (51–56). Sleep-disordered breathing can cause non-restorative sleep, which can in turn affect one's executive functioning (57), vigilance and attentiveness (58), and mood (59). This chain of effects compromises one's academic performance and physical and emotional wellbeing and can cause vulnerability, all of which may lead to CDBs. Thus, sleep problems should also be reviewed as potential triggers for CDBs.

### Creating a New Script for CDBs: The Value of Narratives and Observations

Although our framework of CDBs was developed using rigorous methods, we acknowledge that whether descriptions such as *stubborn, self-stimulating* or *stimming*, and *intrusive* truly differed in scope and could be labeled as *challenging, goofy*, or *unspecified* depended on emotional manifestation (e.g., laughter, violence). Furthermore, the observers' training background and final negotiation or discussion of the context in which behaviors happened will affect labeling. For example, although *being self-conscious* was categorized as a CDB, it may reflect the vulnerability of the participants. Laughter may interrupt the flow of teaching and may be a CDB for the teacher or parents, but not the RAs and reviewing clinicians, both of whom were at arms-length to the incidents.

Extant diagnostic checklists that capture CDBs [e.g., Behavior Problems Inventory (60); Teacher Report Form (61); Nisonger Child Behavior Rating Form—Teacher Version (62); Vanderbilt ADHD Diagnostic Teacher Rating Scale (63)] include many questions with negative connotations. For example, the first item on the Vanderbilt ADHD Diagnostic Teacher Rating Scale asks if the individual “fails to give attention to details or makes careless mistakes in schoolwork” (63). Are “mistakes” not learning opportunities? Likewise, the first item on the Teacher Report Form asks whether the individual “acts too young for his/her age” (61). To whom is the individual compared when considering if they are “too young”? In contrast to these checklists, interestingly all developed in the context of diagnosing behaviors, we suggest an immediate review of CDBs in the situational context and the exploration of the severity and impression of each CDB without clinical diagnostic labeling. A review of CDBs in a non-judgmental exploratory framework (Figure 6) allows one to reflect on the interchangeability of the observers' perceptions and conflicts of interest (e.g., teaching in a quiet environment) and respects the autonomy of the individual subject.

As advocated by Gorman et al. (15), “a primary task for the clinician is to engage the patient and family in a collaborative process to choose among reasonable options, including the option to *forego medication* [emphasis added]” (pp73–74). However, despite the availability of applied behavioral analysis as a non-pharmacological approach to CDBs (16, 64), strategies to manage CDBs rely heavily on stimulant and antipsychotic use, particularly in North American medical practice (65–67). In this context, our explorative approach allows for reflection before medication treatment is considered. The model accommodates the perspectives of the affected individuals and enables a review of the possible explanatory models before any negatively connotated diagnostic labels are made. The recognition of probable familial RLS, which could be partly treated by physical activity, proves that there are many non-pharmacological treatment strategies. This supports a personalized approach to the affected individuals if the root causes of CDBs are recognized. Our grounded, pragmatic approach can be utilized by non-professionals as a beginning step to capture relevant information and to explore CDBs.

### Strengths and Limitations

We collected descriptive, non-judgmental information by non-professionals in a first attempt to create an explorative approach for reviewing CDBs. We used interviews and observations and expanded educational and medical perspectives with therapeutic emplotment, a methodology from medical anthropology. This research builds on our previous work integrating narratives in clinical history-taking (68) and structured behavioral observations (21, 50, 69, 70), and on similar studies that have been undertaken in other clinical populations (71–75). Using such a concept (50) was useful for understanding how CDBs may be interpreted and how observable symptoms of physical activity and sleep (e.g., length, disruptions) may affect CDBs.

We focused on five adolescents with Down syndrome as one example of ID, but our concept is applicable to ID in general irrespective of the etiology and categorical diagnoses. However, we had a small sample size and did not investigate unobservable parts of physical activity and sleep, such as affective responses, the significance of restorative sleep, and restlessness. Although participating families appeared to accept our explanations at the exit interview, we did not follow-up with the participants in a medical setting and cannot report to what extent our explanations were used or implemented by the families. Still, our findings were shared with families and professionals at the DSRF, and all (except for a clinician) agreed with our approach and recommendations.

One other limitation is that we were not able to provide detailed medical data and static psychosocial triggers of the subjects enrolled. In future studies aiming to validate our findings in a larger number of subjects, information about concomitant medications, physical comorbidities, the severity of ID, and psychosocial background should be collected. Finally, the category of *unspecified* behaviors needs further exploration. Play therapy (76, 77) may be an appropriate setting for further characterization of this category.

## Conclusion

In this study, we explored the nature and possible triggers of CDBs of individuals with ID. We focused on five adolescents with Down syndrome, given that they form a cohort with comparable comorbidities. From our interviews and observations, two findings surfaced as significant. The first, and most important, finding was that we should consider our own perceptions and connotations of CDBs in a non-judgmental way, and room for light-hearted behaviors should be created as not all may be truly *challenging* or *disruptive*. The second finding was that physical activity had a visible positive effect on sleep quality and CDBs the following day, suggesting that there may be a biological, in addition to a psychosocial, aspect to CDBs. In summary, this study reminds us that we all need to reflect on our perspectives, to be non-judgmental, and to consider the context in our interpretations. The creation of a new script for CDBs, utilizing *in dubio pro reo*, is a step toward personalized medicine and personal meaningful outcome measures.

## Data Availability Statement

The raw data supporting the conclusions of this article will be made available by the authors, without undue reservation.

## Ethics Statement

The studies involving human participants were reviewed and approved by the University of British Columbia Children's and Women's Research Ethics Board. Written informed consent to participate in this study was provided by the participants' legal guardian/next of kin.

## Author Contributions

MC-HC, MC, SS, and OI contributed to the conception and design of the study. MC-HC, MC, and OI collected the data. MC-HC, MC, and NB analyzed the data. MC-HC and NB developed the data visualizations. MC-HC and OI wrote the manuscript. All authors contributed to manuscript revision and approved the submitted version.

## Funding

This research was supported by a BC Children's Hospital Research Institute Summer Student Scholarship to Mackenzie Campbell in 2016 and 2017, and by a Sleep Research Grant from the BC Children's Hospital to Melvin Chan in 2019. The development of the foundation for a service design concept for community-based data collection and the Sleep/Wake-Behaviour Application were supported by a grant from the Kids Brain Health Network (previously NeuroDevNet) and Telus Community Award 2014.

## Conflict of Interest

The authors declare that the research was conducted in the absence of any commercial or financial relationships that could be construed as a potential conflict of interest.

## Publisher's Note

All claims expressed in this article are solely those of the authors and do not necessarily represent those of their affiliated organizations, or those of the publisher, the editors and the reviewers. Any product that may be evaluated in this article, or claim that may be made by its manufacturer, is not guaranteed or endorsed by the publisher.

## References

[1] Muratori F Santocchi E Calderoni S. Psychiatric assessment. Handb Clin Neurol. (2020) 174:217–38. 10.1016/B978-0-444-64148-9.00016-8 32977880

[2] Simplican SC. Behaviors that challenge disability studies. Disabil Soc. (2019) 34:1379–98. 10.1080/09687599.2018.1552119

[3] Kleinman A Benson P. Anthropology in the clinic: the problem of cultural competency and how to fix it. PLoS Med. (2006) 3:e294. 10.1371/journal.pmed.0030294 17076546 PMC1621088

[4] Emerson E. Challenging Behaviour: Analysis and Intervention in People With Severe Intellectual Disabilities. Cambridge: Cambridge University Press (2001). p. 224.

[5] de Winter CF. Jansen AaC, Evenhuis HM. Physical conditions and challenging behaviour in people with intellectual disability: a systematic review. J Intellect Disabil Res. (2011) 55:675–98. 10.1111/j.1365-2788.2011.01390.x 21366751

[6] Gavidia-Payne S Hudson A. Behavioural supports for parents of children with an intellectual disability and problem behaviours: an overview of the literature. J Intellect Dev Disabil. (2002) 27:31–55. 10.1080/13668250120119626

[7] Emerson E Bromley J. The form and function of challenging behaviours. J Intellect Disabil Res. (1995) 39:388–98. 10.1111/j.1365-2788.1995.tb00543.x 8555715

[8] Soles T Bloom EL Heath NL Karagiannakis A. An exploration of teachers' current perceptions of children with emotional and behavioural difficulties. Emot Behav Diffic. (2008) 13:275–90. 10.1080/13632750802442201

[9] Macleod G. Identifying obstacles to a multidisciplinary understanding of ‘disruptive' behaviour. Emot Behav Diffic. (2010) 15:95–109. 10.1080/13632752.2010.480881

[10] Royal College of Psychiatrists. DC-LD: Diagnostic Criteria for Psychiatric Disorders for Use With Adults With Learning Disabilities/Mental Retardation. London: Gaskell (2001). p. 128.

[11] Byrne A Hennessy E. Understanding challenging behaviour: Perspectives of children and adolescents with a moderate intellectual disability. J Appl Res Intellect Disabil. (2009) 22:317–25. 10.1111/j.1468-3148.2008.00465.x

[12] Clarke A Dagnan D Smith IC. How service-users with intellectual disabilities understand challenging behaviour and approaches to managing it. J Appl Res Intellect Disabil. (2019) 32:1203–15. 10.1111/jar.12612 31066173

[13] Speranzini N Goodarzi Z Casselman L Pringsheim T. Barriers and facilitators associated with the management of aggressive and disruptive behaviour in children: a qualitative study with pediatricians. J Can Acad Child Adolesc Psychiatry. (2020) 29:177–87. 32774400 PMC7391873

[14] Mark TL. For what diagnoses are psychotropic medications being prescribed? CNS Drugs. (2010) 24:319–26. 10.2165/11533120-000000000-00000 20297856

[15] Gorman DA Gardner DM Murphy AL Feldman M. Bélanger SA, Steele MM, et al. Canadian guidelines on pharmacotherapy for disruptive and aggressive behaviour in children and adolescents with attention-deficit hyperactivity disorder, oppositional defiant disorder, or conduct disorder. Can J Psychiatry. (2015) 60:62–76. 10.1177/070674371506000204 25886657 PMC4344948

[16] Grey IM Hastings RP. Evidence-based practices in intellectual disability and behaviour disorders. Curr Opin Psychiatry. (2005) 18:469–275. 10.1097/01.yco.0000179482.54767.cf 16639103

[17] Heyvaert M Maes B Onghena P. A meta-analysis of intervention effects on challenging behaviour among persons with intellectual disabilities. J Intellect Disabil Res. (2010) 54:634–49. 10.1111/j.1365-2788.2010.01291.x 20492347

[18] Lory C Mason RA Davis JL Wang D Kim SY Gregori E. A meta analysis of challenging behavior interventions for students with developmental disabilities in inclusive school settings. J Autism Dev Disord. (2020) 50:1221–37. 10.1007/s10803-019-04329-x 31907730

[19] Michail S. Understanding school responses to students' challenging behaviour: a review of literature. Improv Sch. (2011) 14:156–71. 10.1177/1365480211407764

[20] Walker VL Carpenter ME Lyon KJ Button L. A meta-analysis of paraprofessional-delivered interventions to address challenging behavior among students with disabilities. J Posit Behav Interv. (2021) 23:68–80. 10.1177/1098300720911147

[21] Ipsiroglu OS. Applying Ethnographic Methodologies & Ecology to Unveil Dimensions of Sleep Problems in Children & Youth With Neurodevelopmental Conditions. Vancouver, BC: The University of British Columbia (2016). p. 249.

[22] Beard H. Latin for all occasions: From cocktail-party banter to climbing the corporate ladder to online dating—Everything you'll ever need to say in perfect Latin. Avery; 2004. 176 p.

[23] Bull MJ Committee Committee on Genetics. Health supervision for children with Down syndrome. Pediatrics. (2011) 128:393–406. 10.1542/peds.2011-1605 21788214

[24] Dykens EM. Psychiatric and behavioral disorders in persons with Down syndrome. Ment Retard Dev Disabil Res Rev. (2007) 13:272–8. 10.1002/mrdd.20159 17910080

[25] Roizen NJ Patterson D. Down's syndrome. Lancet. (2003) 361:1281–9. 10.1016/S0140-6736(03)12987-X 12699967

[26] Patel L Wolter-Warmerdam K Leifer N Hickey F. Behavioral characteristics of individuals with Down syndrome. J Ment Health Res Intellect Disabil. (2018) 11:221–46. 10.1080/19315864.2018.1481473

[27] Dieleman LM De Pauw Sarah SW Soenens B Van Hove G Prinzie P. Behavioral problems and psychosocial strengths: unique factors contributing to the behavioral profile of youth with Down syndrome Am J Intellect Dev Disabil. (2018) 123:212–27. 10.1352/1944-7558-123.3.212 29671633

[28] Patel L Wolter-Warmerdam K Hickey F. Patterns of behavior and medical comorbidities in Down syndrome. J Ment Health Res Intellect Disabil. (2020) 13:267–80. 10.1080/19315864.2020.1790064

[29] Hauser-Cram P Warfield ME Shonkoff JP Krauss MW Upshur CC Sayer A. Family influences on adaptive development in young children with Down syndrome. Child Dev. (1999) 70:979–89. 10.1111/1467-8624.00071 10446730

[30] Huiracocha L Almeida C Huiracocha K Arteaga J Arteaga A Blume S. Parenting children with Down syndrome: societal influences. J Child Health Care. (2017) 21:488–97. 10.1177/1367493517727131 29110530 PMC5697561

[31] Van Hooste A Maes B. Family factors in the early development of children with Down syndrome. J Early Interv. (2003) 25:296–309. 10.1177/105381510302500405

[32] Eccles JS Roeser RW. School and community influences on human development. In: Bornstein MH, Lamb ME, editors. Developmental Science: An Advanced Textbook. London: Psychology Press (2011). p. 571–643.

[33] Down, Syndrome Resource Foundation. Our Mission and Vision. Available online at: https://www.dsrf.org/about-us/about-dsrf/ (accessed January 9, 2021).

[34] Bijou SW Peterson RF Ault MH. A method to integrate descriptive and experimental field studies at the level of data and empirical concepts. J Appl Behav Anal. (1968) 1:175–91. 10.1901/jaba.1968.1-175 16795175 PMC1310995

[35] Kleinman A. The Illness Narratives: Suffering, Healing, and the Human Condition. New York, NY: Basic Books (1988). p. 304.

[36] Mattingly C. The concept of therapeutic “emplotment”. Soc Sci Med. (1994) 38:811–22. 10.1016/0277-9536(94)90153-8 8184332

[37] Frank G. “Becoming the other”: empathy and biographical interpretation. Biography. (1985) 8:189–210. 10.1353/bio.2010.0479

[38] Heng TB Gupta A Shaw C Raber C Schilling M Chen N. Sleep-wake-behaviour app: toward developing a database for informing e-coaching solutions for neurodevelopmental disorders in children. In: PervasiveHealth '18: Proceedings of the 12th EAI International Conference on Pervasive Computing Technologies for Healthcare. New York, NY (2018). p. 371–7.

[39] Venn J. Symbolic Logic. London: Macmillan and Company (1881). p. 446.

[40] Nunkoosing K Haydon-Laurelut M. Intellectual disability trouble: foucault and Goffman on ‘challenging behaviour'. In: Goodley D, Hughes B, Davis L, editors. Disability and Social Theory: New Developments and Directions. London: Palgrave Macmillan (2012). p. 195–211.

[41] Lyons CW O'Connor F. Constructing an integrated model of the nature of challenging behaviour: a starting point for intervention. Emot Behav Diffic. (2006) 11:217–32. 10.1080/13632750600833973

[42] Silverman W. Down syndrome: cognitive phenotype. Ment Retard Dev Disabil Res Rev. (2007) 13:228–36. 10.1002/mrdd.20156 17910084

[43] Lloyd BP Kennedy CH. Assessment and treatment of challenging behaviour for individuals with intellectual disability: a research review. J Appl Res Intellect Disabil. (2014) 27:187–99. 10.1111/jar.12089 24464965

[44] Feeley K Jones E. Addressing challenging behaviour in children with Down syndrome: The use of applied behaviour analysis for assessment and intervention. Downs Syndr Res Pract. (2006) 11:64–77. 10.3104/perspectives.316 17048800

[45] Bruni M Cameron D Dua S Noy S. Reported sensory processing of children with Down syndrome. Phys Occup Ther Pediatr. (2010) 30:280–93. 10.3109/01942638.2010.486962 20735195

[46] Will EA Daunhauer LA Fidler DJ Raitano Lee N Rosenberg CR Hepburn SL. Sensory processing and maladaptive behavior: profiles within the Down syndrome phenotype. Phys Occup Ther Pediatr. (2019) 39:461–76. 10.1080/01942638.2019.1575320 31070074 PMC8011957

[47] Savage BM Lujan HL Thipparthi RR DiCarlo SE. Humor, laughter, learning, and health! A brief review. Adv Physiol Educ. (2017) 41:341–7. 10.1152/advan.00030.2017 28679569

[48] Salma B-A Yanping L De VK Atul M John W Xiang G. Lifestyle factors and risk of restless legs syndrome: prospective cohort study. J Clin Sleep Med. (2016) 12:187–94. 10.5664/jcsm.5482 26446243 PMC4751426

[49] Aukerman MM Aukerman D Bayard M Tudiver F Thorp L Bailey B. Exercise and restless legs syndrome: a randomized controlled trial. J Am Board Fam Med. (2006) 19:487–93. 10.3122/jabfm.19.5.487 16951298

[50] Ipsiroglu OS Beyzaei N Berger M Wagner AL Dhalla S Garden J. “Emplotted narratives” and structured “behavioral observations” supporting the diagnosis of Willis-Ekbom disease/restless legs syndrome in children with neurodevelopmental conditions. CNS Neurosci Ther. (2016) 22:894–905. 10.1111/cns.12564 27292821 PMC5095767

[51] Churchill SS Kieckhefer GM Bjornson KF Herting JR. Relationship between sleep disturbance and functional outcomes in daily life habits of children with Down syndrome. Sleep. (2015) 38:61–71. 10.5665/sleep.4326 25325444 PMC4262957

[52] Esbensen AJ Hoffman EK Beebe DW Byars KC Epstein J. Links between sleep and daytime behaviour problems in children with Down syndrome. J Intellect Disabil Res. (2018) 62:115–25. 10.1111/jir.12463 29282827 PMC5775033

[53] Horne RSC Wijayaratne P Nixon GM Walter LM. Sleep and sleep disordered breathing in children with down syndrome: Effects on behaviour, neurocognition and the cardiovascular system. Sleep Med Rev. (2019) 44:1–11. 10.1016/j.smrv.2018.11.002 30576943

[54] Stores RJ. A preliminary study of sleep disorders and daytime behaviour problems in children with Down syndrome. Downs Syndr Res Pract. (1993) 1:29–33. 10.3104/reports.826521721

[55] Stores RJ Stores G Fellows B Buckley S. A factor analysis of sleep problems and their psychological associations in children with Down's syndrome. J Appl Res Intellect Disabil. (1998) 11:345–54. 10.1111/j.1468-3148.1998.tb00042.x

[56] Stores RJ Stores G. The significance of aspects of screening for obstructive sleep apnoea in children with Down syndrome. J Intellect Disabil Res. (2014) 58:381–92. 10.1111/jir.12033 23489956

[57] Turnbull K Reid GJ Morton JB. Behavioral sleep problems and their potential impact on developing executive function in children. Sleep. (2013) 36:1077–84. 10.5665/sleep.2814 23814345 PMC3669074

[58] de Bruin EJ van Run C Staaks J Meijer AM. Effects of sleep manipulation on cognitive functioning of adolescents: A systematic review. Sleep Med Rev. (2017) 32:45–57. 10.1016/j.smrv.2016.02.006 27039223

[59] Asarnow LD Mirchandaney R. Sleep and mood disorders among youth. Child Adolesc Psychiatr Clin N Am. (2021) 30:251–68. 10.1016/j.chc.2020.09.003 33223065 PMC8386498

[60] Rojahn J Rowe EW Sharber AC Hastings R Matson JL Didden R. The behavior problems inventory-short form for individuals with intellectual disabilities: Part I: development and provisional clinical reference data. J Intellect Disabil Res. (2012) 56:527–45. 10.1111/j.1365-2788.2011.01507.x 22151184

[61] Achenbach TM Rescorla LA. Manual for the ASEBA School-Age Forms & Profiles. Burlington: University of Vermont, Research Center for Children, Youth, and Families (2001). p. 238.

[62] Aman MG Tassé MJ Rojahn J Hammer D. The Nisonger CBRF: A child behavior rating form for children with developmental disabilities. Res Dev Disabil. (1996) 17:41–57. 10.1016/0891-4222(95)00039-9 8750075

[63] Wolraich ML Feurer ID Hannah JN Baumgaertel A Pinnock TY. Obtaining systematic teacher reports of disruptive behavior disorders utilizing DSM-IV. J Abnorm Child Psychol. (1998) 26:141–52. 10.1023/A:1022673906401 9634136

[64] Newcomb ET Hagopian LP. Treatment of severe problem behaviour in children with autism spectrum disorder and intellectual disabilities. Int Rev Psychiatry. (2018) 30:96–109. 10.1080/09540261.2018.1435513 29537889 PMC8793042

[65] Zhang T Smith MA Camp PG Shajari S MacLeod SM Carleton BC. Prescription drug dispensing profiles for one million children: a population-based analysis. Eur J Clin Pharmacol. (2013) 69:581–8. 10.1007/s00228-012-1343-1 22791273

[66] Epstein RA Fonnesbeck C Potter S Rizzone KH McPheeters M. Psychosocial interventions for child disruptive behaviors: a meta-analysis. Pediatrics. (2015) 136:947–60. 10.1542/peds.2015-2577 26482672

[67] Pringsheim T Stewart DG Chan P Tehrani A Patten SB. The pharmacoepidemiology of psychotropic medication use in Canadian children from 2012 to 2016. J Child Adolesc Psychopharmacol. (2019) 29:740–5. 10.1089/cap.2019.0018 31355670

[68] Ipsiroglu OS Jan JE Freeman RD Laswick AJ Milner RA Mitton C. How to approach pediatric sleep medicine in British Columbia: a consensus paper. B C Med J. (2008) 50:512–6.

[69] Beyzaei N Bao S Bu Y Hung L Hussaina H Maher KS. Is Fidgety Philip's ground truth also ours? The creation and application of a machine learning algorithm. J Psychiatr Res. (2020) 131:144–51. 10.1016/j.jpsychires.2020.08.033 32971358

[70] Chan M Tse EK Bao S Berger M Beyzaei N Campbell M. Fidgety philip and the suggested clinical immobilization test: annotation data for developing a machine learning algorithm. Data in Brief. (2021) 35:106770. 10.1016/j.dib.2021.106770 33553523 PMC7851356

[71] Adams HL Matson JL Jang J. The relationship between sleep problems and challenging behavior among children and adolescents with autism spectrum disorder. Res Autism Spectr Disord. (2014) 8:1024–30. 10.1016/j.rasd.2014.05.00834365886

[72] Brylewski J Wiggs L. Sleep problems and daytime challenging behaviour in a community-based sample of adults with intellectual disability. J Intellect Disabil Res. (1999) 43:504–12. 10.1046/j.1365-2788.1999.00234.x 10622367

[73] Ipsiroglu OS McKellin WH Carey N Loock C. “They silently live in terror…” why sleep problems and night-time related quality-of-life are missed in children with a fetal alcohol spectrum disorder. Soc Sci Med. (2013) 79:76–83. 10.1016/j.socscimed.2012.10.027 23305724

[74] Rzepecka H McKenzie K McClure I Murphy S. Sleep, anxiety and challenging behaviour in children with intellectual disability and/or autism spectrum disorder. Res Dev Disabil. (2011) 32:2758–66. 10.1016/j.ridd.2011.05.034 21700417

[75] Wiggs L Stores G. Severe sleep disturbance and daytime challenging behaviour in children with severe learning disabilities. J Intellect Disabil Res. (1996) 40:518–28. 10.1111/j.1365-2788.1996.tb00662.x 9004112

[76] Astramovich RL Lyons C Hamilton NJ. Play therapy for children with intellectual disabilities. J Child Adolesc Ment Health. (2015) 1:27–36. 10.1080/23727810.2015.1015904

[77] Axline VM. Play Therapy; The Inner Dynamics of Childhood. Boston: Houghton Mifflin (1947). p. 379.

